# Identification of Genes Related to Hair Follicle Cycle Development in Inner Mongolia Cashmere Goat by WGCNA

**DOI:** 10.3389/fvets.2022.894380

**Published:** 2022-06-14

**Authors:** Gao Gong, Yixing Fan, Xiaochun Yan, Wenze Li, Xiaomin Yan, Hongfu Liu, Ludan Zhang, Yixing Su, Jiaxin Zhang, Wei Jiang, Zhihong Liu, Zhiying Wang, Ruijun Wang, Yanjun Zhang, Qi Lv, Jinquan Li, Rui Su

**Affiliations:** ^1^College of Animal Science, Inner Mongolia Agricultural University, Hohhot, China; ^2^College of Animal Science and Veterinary Medicine, Shenyang Agricultural University, Shenyang, China; ^3^Key Laboratory of Animal Genetics, Breeding and Reproduction, Inner Mongolia Agricultural University, Hohhot, China; ^4^Key Laboratory of Mutton Sheep Genetics and Breeding, Ministry of Agriculture and Rural Affairs, Hohhot, China; ^5^Engineering Research Center for Goat Genetics and Breeding, Hohhot, China

**Keywords:** hair follicle cycle, WGCNA, core genes, Inner Mongolia cashmere goats, Wnt signaling pathway

## Abstract

Cashmere goat from Inner Mongolia is an excellent local breed in China, and the related cashmere product is a kind of precious textile raw material with high price. Cashmere is generated from secondary hair follicles, which has obvious annual periodicity and includes three different stages: anagen, catagen, and telogen. Therefore, we investigated skin transcriptome data for 12 months using weighted gene co-expression network analysis (WGCNA) to explore essential modules, pathways, and genes responsible for the periodic growth and development of secondary hair follicles. A total of 17 co-expression modules were discovered by WGCNA, and there is a strong correlation between steelblue module and month (0.65, *p* = 3E−09), anagen (0.52, *p* = 1E−05), telogen (−0.6, *p* = 8E−08). Gene expression was generally high during late anagen to catagen (June to December), while expression was downregulated from telogen to early anagen (January–May), which is similar to the growth rule of hair follicle cycle. KEGG pathway enrichment analyses of the genes of steelblue module indicated that genes are mainly enriched in Cell cycle, Wnt signaling pathway, p53 signaling pathway and other important signal pathways. These genes were also significantly enriched in GO functional annotation of the cell cycle, microtubule movement, microtubule binding, tubulin binding, and so on. Ten genes (*WIF1, WNT11, BAMBI, FZD10, NKD1, LEF1, CCND3, E2F3, CDC6*, and *CDC25A*) were selected from these modules, and further identified as candidate biomarkers to regulate periodic development of hair follicles using qRT-PCR. The Wnt signaling pathway and Cell cycle play an important role in the periodic development of hair follicles. Ten genes were identified as essential functional molecules related to periodic development of hair follicle. These findings laid a foundation for understanding molecular mechanisms in biological functions such as hair follicle development and hair growth in cashmere goats.

## Introduction

**Figure 1:**
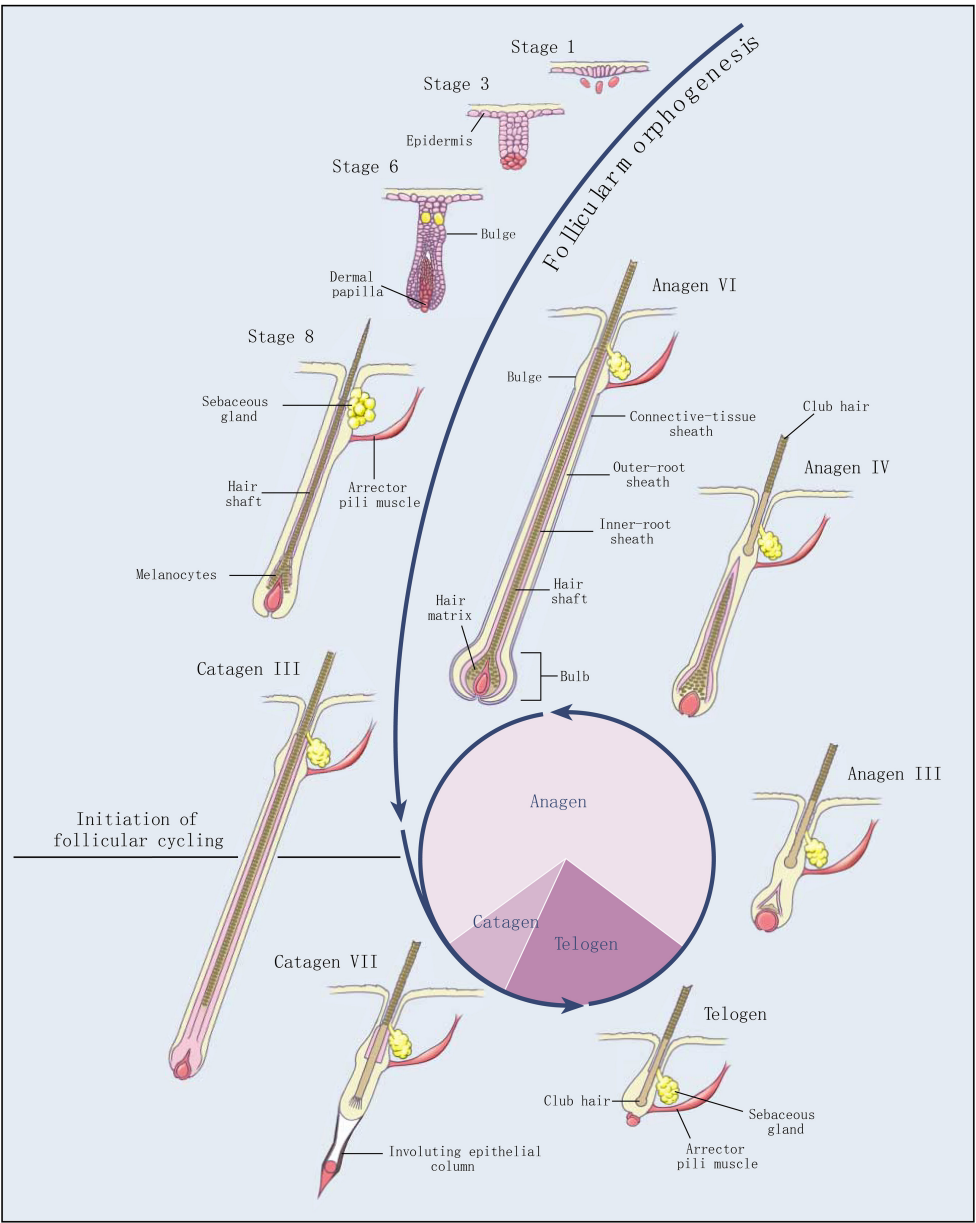
Development and cycling of hair follicles (2). Selected stages of the morphogenesis of hair follicles and the three stages of follicular cycling (anagen, catagen, and telogen) are shown. The roman numerals indicate morphologic substages of anagen and catagen. The pie chart shows the proportion of time the hair follicle spends in each stage.

Cashmere goat is a typical economic breed, providing high quality meat and cashmere for human beings. The coat obtained from the cashmere goat is a typical heterogeneous fleece, including both myelinated coarse hairs generated by primary hair follicle (PHF) and unmyelinated cashmere generated by secondary hair follicle (SHF). Cashmere has significant advances in softness, smoothness, slenderness, lightness, elasticity, heat retention, comfort, etc. Therefore, cashmere is known as “fiber gemstone” or “soft gold,” and is the best natural fiber raw material in the textile industry (1). Hair follicles of cashmere goats exhibit an obvious growth cycle, including anagen, catagen, and telogen (Figure 1) (2). Like a tiny organ of hair growth, hair follicles are regulated by various factors, such as heredity, nutrition, season, age, shearing, environment, and so on.

The secondary hair follicles of Inner Mongolia cashmere goat have a typical cycle, whose shed season is one year, from April to May every year. The growth cycle includes anagen (April–November), catagen (December–January of the following year), and telogen (February–March) (3, 4). During the telogen, the epithelial bud cells at the bottom of the secondary hair follicles begin to extend downward, and the hair papillae are activated to start hair follicle reconstruction. Hair follicles enter the anagen and begin to grow rapidly. After entering the catagen phase, the hair balls begin to atrophy and become thinner, and gradually move upward; the hair follicles entering the telogen are only above the sebaceous glands, and the cells are in a static state (5). The periodic growth of cashmere goat hair follicles is regulated by a series of molecules, including hormone regulation, such as melatonin (6), prolactin (7), thyroxine (8), and so on. Growth factors are also involved in the regulation of hair follicles, including IGFs (9), FGFs (10), VEGF (11), PDGF (12), and so on. In addition, Keratin genes (*KRTs*) (13), Keratin-associated proteins (*KAPs*) (14), and bone morphogenetic proteins (*BMPs*) (15) are proven to regulate hair follicles. Signaling pathways, including the Wnt signaling pathway (16), PI3K-Akt signaling pathway (17), MAPK signaling pathway (18), Ras signaling pathway (19), cell cycle pathway (13), and so on also play important roles in the development of hair follicle (20).

In recent years, with the development of life science, sequencing technology and other high-throughput methods have been well developed. As the cost of high-throughput sequencing is decreasing, a large amount of sequencing data is gradually cumulated. Weighted gene co-expression network analysis (WGCNA) is a useful tool to analyze these high-throughput data (21–23). WGCNA has great advances in understanding the molecular mechanisms of complex traits and diseases such as cell cycle (21), lung cancer (24), pancreatic cancer (25), including genetic diseases in humans, mice, and many other organisms. This method combines high-throughput data with sample phenotypic data for joint analysis to define a small number of modules and identify key markers, which greatly improve the analytical efficiency of high-throughput data (22). The genetic mechanisms of cashmere quality traits can be further analyzed by the WGCNA method. Recently, some researchers applied WGCNA to analyze the hair follicle development and identified the module consistent with the embryonic hair follicle development stage of Inner Mongolia cashmere goat, as well as the key gene (*WNT10A*) for the mature stage of hair follicle development in Inner Mongolia cashmere goat skin (26). Many researchers have found that ECM-receptor interaction, Focal adhesion, PI3K-Akt signaling pathway, Estrogen signaling pathway, Wnt signaling pathway, Hedgehog signaling pathway, Cell cycle, Arachidonic acid metabolism, cAMP signaling pathway, and other signal pathways are closely related to the periodic growth of hair follicles. The core genes such as *COL1A1, C1QTNF6, KRTAP3-1, DLX3, OVOL1, COL1A1, C1QTNF6, KRTAP3-1, WNT5A, LOC102172600*, and *LOC102191729* were screened (13, 27–30). In this study, we used WGCNA to analyze the gene expression data of 12 months from Inner Mongolia cashmere goats, and identify important modules and genes related to the periodic growth of secondary hair follicles. The genes were further validated by GO, KEGG, gene connectivity network, and real-time fluorescence quantitative technique. This study provides a new insight into the regulation mechanism of periodic growth of hair follicles in cashmere goats.

## Materials and Methods

### Data Sources

In this study, we used transcriptome data that our team (SRA: PRJNA832904). The sequenced individuals were six 2-year-old adult ewes of unrelated Inner Mongolia cashmere goats (Alba type). The skin tissues were collected once a month for 12 months. RNA-seq was carried out by using Illumina X Ten sequencing platform system. Sequence alignment was carried out with the goat genome (*Capra hircus*, ARS1) by HISAT (2.0.4) (31). The gene expression level of each sample was analyzed by HTSeq (v0.6.1) (32), and the gene expression data (FPKM) was calculated. The genes with zero expression in more than 16 samples were filtered, and the differential expression of genes between the two groups was analyzed by DESeq (*p_adj_* < 0.05) (33), and all the differential genes were merged. The filtered dataset contains 7,320 gene expression data from 72 samples. The skin sample in January was marked as M_1 (_1, _2, _3, _4, _5, _6).

### Sample Collection

In this study, skin samples were collected in accordance with the International Guiding Principles for Biomedical Research Involving Animals and were approved by the Animal Ethics Committee of the Inner Mongolia Academy of Agriculture and Animal Husbandry Sciences which is responsible for animal care and use in the Inner Mongolia Autonomous Region of China. The experimental animals came from Inner Mongolia Jin Lai Livestock Technology Company (Hohhot, Inner Mongolia, China). All Inner Mongolia cashmere goats are raised according to the standard of cashmere goats. The skin tissues of 3 Albus adult ewes of Inner Mongolia cashmere goat were collected for 12 months. The sampling site was the upper one-third of the left scapula along the mid-dorsal and mid-abdominal lines. About 1% pentobarbital sodium anesthetic was used for muscle anesthesia. After hair shearing and alcohol disinfection, ~1 cm^2^ of skin tissue was grasped with sterile forceps and quickly cut near the tip using sterile scalpel blades. Each clipping was obtained immediately adjacent to the location of the previous shearing. Yunnan Baiyao powder (Yunnan Baiyao Group Co., Ltd., China) was applied immediately to stop the bleeding. Then the samples were quickly put into the liquid nitrogen and finally stored at −80°C. Skin samples were used for RNA extraction and quantitative real-time PCR.

### Weighted Gene Coexpression Network Analysis

The hair follicle cycle development of Inner Mongolia cashmere goat was analyzed by weighted gene co-expression network analysis (WGCNA, Version 1.70-3) in R (Version 4.1.0) (21). The specific usage of the R package was referred to the official website of WGCNA (https://horvath.genetics.ucla.edu/html/CoexpressionNetwork/Rpackages/WGCNA/) (22). Firstly, based on the correlations between samples, a clustering tree was drawn to eliminate outliers (h > 16,000). Using the gene expression data, the absolute value of the expression correlation coefficient between the two genes was calculated, and the gene co-expression correlation matrix (*S_ij_* = |*cor*(*X_i_*, *X_j_*)|) was constructed. In order to realize the scale-free network, the adjacency degree β between genes is calculated by pickSoftThreshold function, and the β value of R^2^ > 0.8 was selected to construct the adjacency matrix ($a_{ij}\;=\;S_{ij}^{\beta }$). Finally, in order to evaluate the correlation between genes, a topological overlap matrix [$TOM_{ij}\;=\;\frac{\underset{u}{\sum }a_{iu}a_{uj}+a_{ij}}{\min\left\{K_{i},K_{j}\right\}+1-a_{ij}}\left(u\neq i,j\right)$] was constructed. We set the minimum module size to 30, drew the TOM clustering tree by TOM matrix, and the genes with similar expression patterns were clustered into one group through the TOM clustering tree. Merging of modules whose expression profiles were very similar. We choose a Height cutoff set to 0.30, and the corresponding correlation was set to 0.70 (MEDissThres = 0.3). Key gene modules were identified by associating with phenotypic. Different developmental stages of the cashmere goat hair follicle cycle were divided by the month of sample collection. April to November was defined as anagen, December to January was defined as catagen, and February to March was defined as telogen to construct a phenotypic matrix. In order to explore the correlations between modules and traits, module-trait relationships were calculated. A heat map of the expression of the key modules was drawn. Through the correlations between module genes and traits, the key modules related to the hair follicle cycle of cashmere goats were identified.

### Core Module Gene Analysis

Kyoto Encyclopedia of Genes and Genomes (KEGG) (34) is a collection of databases dealing with genomes, biological pathways, diseases, drugs, and chemical substances. The Gene Ontology (GO) (35) is a major bioinformatics initiative to unify the representation of gene and gene product attributes across all species. The GO covers three domains: cellular component, molecular function, and biological process. KEGG and GO enrichment analysis of genes of module genes was implemented by the clusterProfiler (4.2.2) (36) R package, in which gene length bias was corrected. The enrichment was considered to be significant when the corrected *p-values* were <0.05. The exportNetworkToCytoscape function in WGCNA is used to derive the network relationship (threshold = 0.10). The output module nodes file and the module edges file were imported into the Cytoscape3.9.0 (37) for network visualization. Combined with the results of weight network and functional enrichment analysis, the candidate was identified, and the gene expression curve was drawn by GraphPad Prism8.3.0 using FPKM data.

### Extraction of Total RNA and Design of Primers

The total RNA of the skin was extracted according to the user guide of TRLzol Reagent (Invitrogen). The concentration and quality of total RNA were determined by NanoDrop 2000 (Thermo). The total RNA that passed the test was synthesized cDNA using PrimeScript synthesis RT reagent Kit with gDNA Eraser (RR047A, TAKARA).

The mRNA coding region sequence of the genes was queried by the NCBI database, and the fluorescent quantitative specific primers of the gene were designed by Primer-BLAST (NCBI) (38). The primer parameters were set as follows: PCR product size 90–200 bp, primer size 18–22 bp, primer melting temperatures (Tm) set to 58.0–62.0°C, primer GC content 40–60%, and other parameters as default values. The primer sequence is shown in Supplementary Table 1, and the primer was handed over to Sangon Biotech (Shanghai) Co., Ltd.

### Quantitative Real-Time PCR

In this study, LightCycler ®96 (Roche) instrument and TB Green “Premix Ex Taq” II (RR820A, TAKARA) kit were used for the Quantitative real-time PCR (qRT-PCR) test. The reaction system is TB Green Premix Ex Taq II (Tli RNaseH Plus) (2×) 5 μl, cDNA 1 μl, PCR Forward/Reverse Primer (10 μM) 0.5 μl, ddH_2_O 3 μl. Programs: Preincubation 95°C 30 s, two step amplification (95°C 5 s, Tm 30 s) for a total of 45 cycles, melting, and cooling. Each month tested three samples and made three technical repeats.

### Statistical Analysis

Excel 2019 was used to collate and summarize the CQ value. The genes were calibrated by *GAPDH* and β*-actin* as double house-keeping genes. The 2^−ΔΔ^CT method (39) was used to calculate the relative gene expression. SAS 9.2 ANOVA was used to analyze the variance of the data. Duncan method was used for multiple comparisons, and the relative gene expression map was drawn by GraphPad Prism 8.3.0. *p* < 0.05 was considered to be statistically significant.

## Results

### Construction of Weighted Gene Co-expression Network

**Figure 2:**
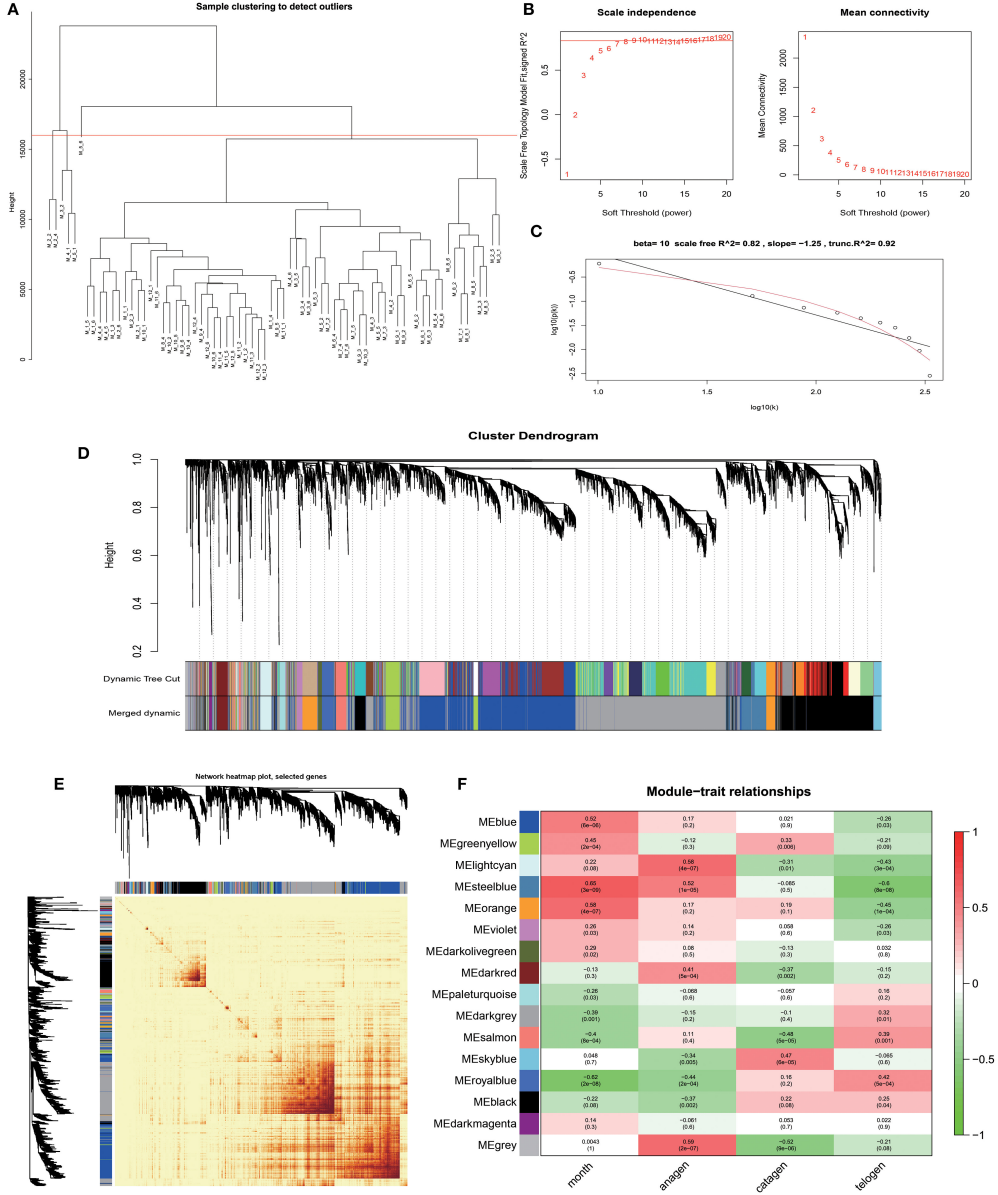
Identification of co-expression modules by WGCNA. **(A)** Hierarchical clustering information of samples, red line = 16,000. **(B)** The determination of soft thresholding power. **(C)** When β = 10, the scale-free network fitting. **(D)** The gene clustering dendrogram was obtained according to hierarchic clustering of adjacency based dissimilarity. **(E)** Network heatmap of module-genes, Each tree represents a module, each branch represents a gene, and the darker the color of each dot, the stronger the connectivity between the two genes corresponding to the row and column. **(F)** Module–trait relationships, Abscissa is the trait, the ordinate is the module, the number of each grid represents the correlation between the module and the trait, and the number in parentheses represents *p*-value.

Based on the gene expression data of skin tissues of Inner Mongolia cashmere goats in different months, we obtained a gene expression matrix including 72 samples and 7,320 genes after filtering. among them. Construct a co-expression network through R packets of WGCNA. The hclust function is used to analyze the samples (Figure 2A). Six outlier samples are found in the hierarchical clustering of the samples and were eliminated in further analyses. In order to build a network with scale-free distribution and retain information as much as possible, a soft threshold method is used to find the best soft-thresholding powers β (Figure 2B). Specific Powervalue information is shown in Supplementary Table 2. We discovered that the connectivity between genes in the network is high (β = 10, R^2^ = 1.82), indicating a scale-free network (Figure 2C). The TOM clustering tree is constructed to divide modules preliminarily, and then similar modules are combined by a dynamic hybrid cutting method (Figure 2D). Finally, genes are divided into 17 modules (Table 1). The largest module contains 2,152 genes (darkgrey module), while the smallest module contains 35 genes (darkmagenta module). The module gene clustering heat map is drawn by hclust function, which is used to show the connectivity relationship between the two genes (Figure 2E). From the module-trait correlation heat map (Figure 2F), we discovered four modules significant related to months and different stages. Steelblue module is significantly correlated with month (0.65, *p* = 3E−09), anagen (0.52, *p* = 1E−05) and telogen (−0.6, *p* = 8E−08), respectively. Royalblue module have strong correlations with month (−0.62, *p* = 2E−08), anagen (−0.44, *p* = 2E−04) and telogen (−0.42, *p* = 5E−04). Lightcyan module has strong correlations with anagen (0.58, *p* = 4E−07) and telogen (−0.43, *p* = 3E−04), while orange module is correlated with month (0.58, *p* = 4E−07). Genes in these modules might be involved in the periodic development of secondary hair follicles.

**Table 1:** Gene number in 17 modules.

**Module color**	**Gene numbers**	**Module color**	**Gene numbers**
Black	1,239	Lightcyan	141
Blue	1,933	Orange	289
Darkgrey	2,152	Paleturquoise	68
Darkmagenta	35	Royalblue	128
Darkolivegreen	44	Salmon	169
Greenyellow	102	Skyblue	86
Darkred	126	Steelblue	348
Greenyellow	282	Violet	63
Grey	217		

### Key Modules Related to Hair Follicle Cycle Development

**Figure 3:**
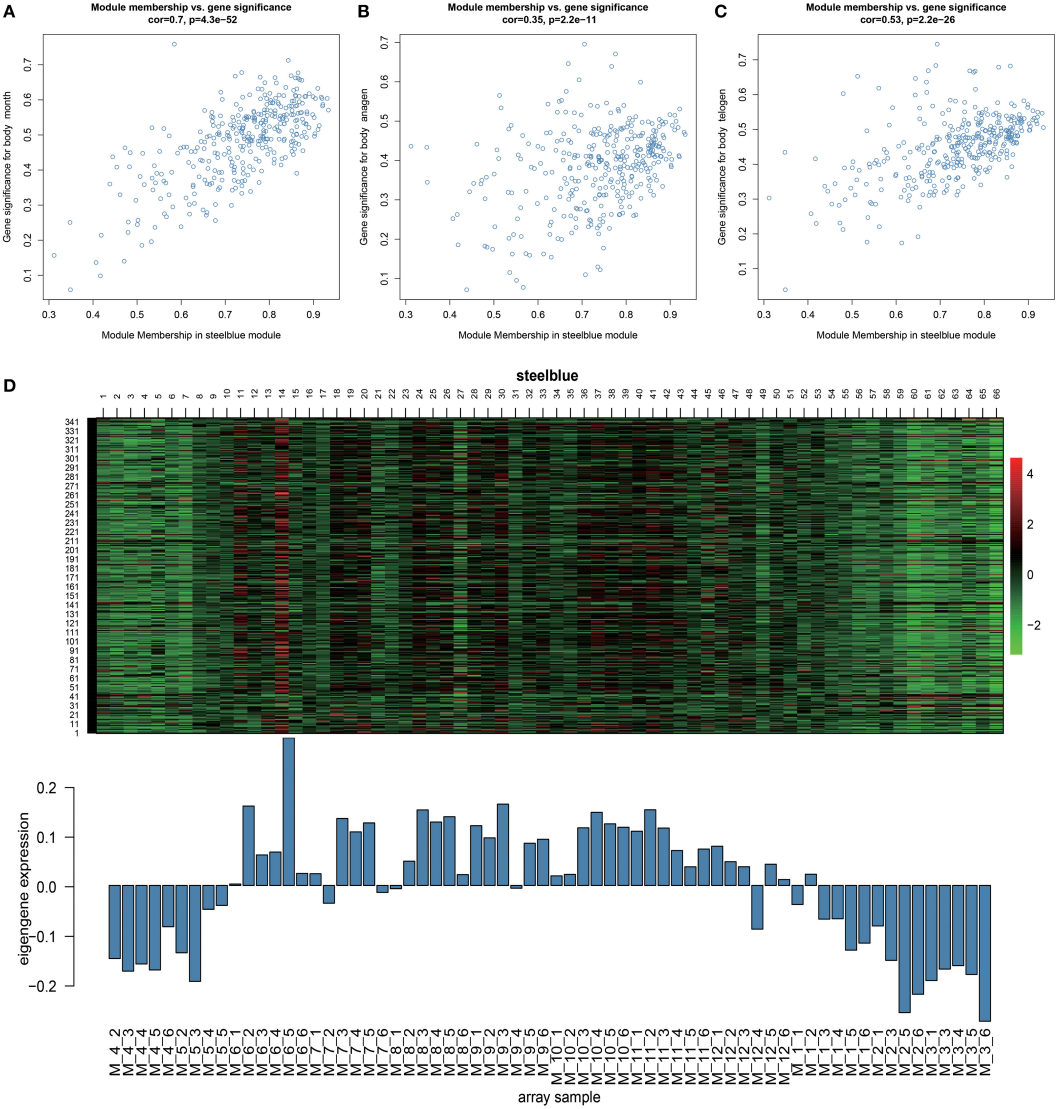
Key module analysis. **(A–C)** Scatterplot of Gene Significance for Module Membership in steelblue module. **(D)** Heatmap of steelblue module genes expression pattern. The Abscissa is the sample name, the above picture is the heat map of the expression of the genes in the module in different samples, and the following picture is the expression pattern of the characteristic values of the module in different samples.

Through Module trait relationships analysis, we found that the steelblue module has the strongest correlations with many stages of hair follicle cycle development. Therefore, we will focus on the genes in this module for follow-up analysis. With a scatter plot of steelblue module's module membership and month, anagen, and telogen's gene significance (Figures 3A–C). We identified a high correlation between steelblue module and month (cor = 0.7, *p* = 4.3E−52), indicating that the gene of this module has a strong correlation with monthly changes. In addition, this steelblue module also has moderate correlations with anagen (cor = 0.35, *p* = 2.2E−11) and telogen (cor = 0.53, *p* = 2.2E−26). These results indicate that genes in this module are strongly associated with the periodic development of hair follicles. There are 348 genes in the steelblue module, including 322 annotated genes and 26 new transcripts (Supplementary Table 3). Then we analyzed steelblue module gene expression patterns in detail (Figure 3D), and discovered specific expression patterns in different months. During anagen, the gene expressions of this module were generally low from April to May and high from June to November. During catagen, the expressions began to decrease. During telogen, the gene expressions were generally low.

### Functional Annotations of Steelblue Module Genes

**Figure 4:**
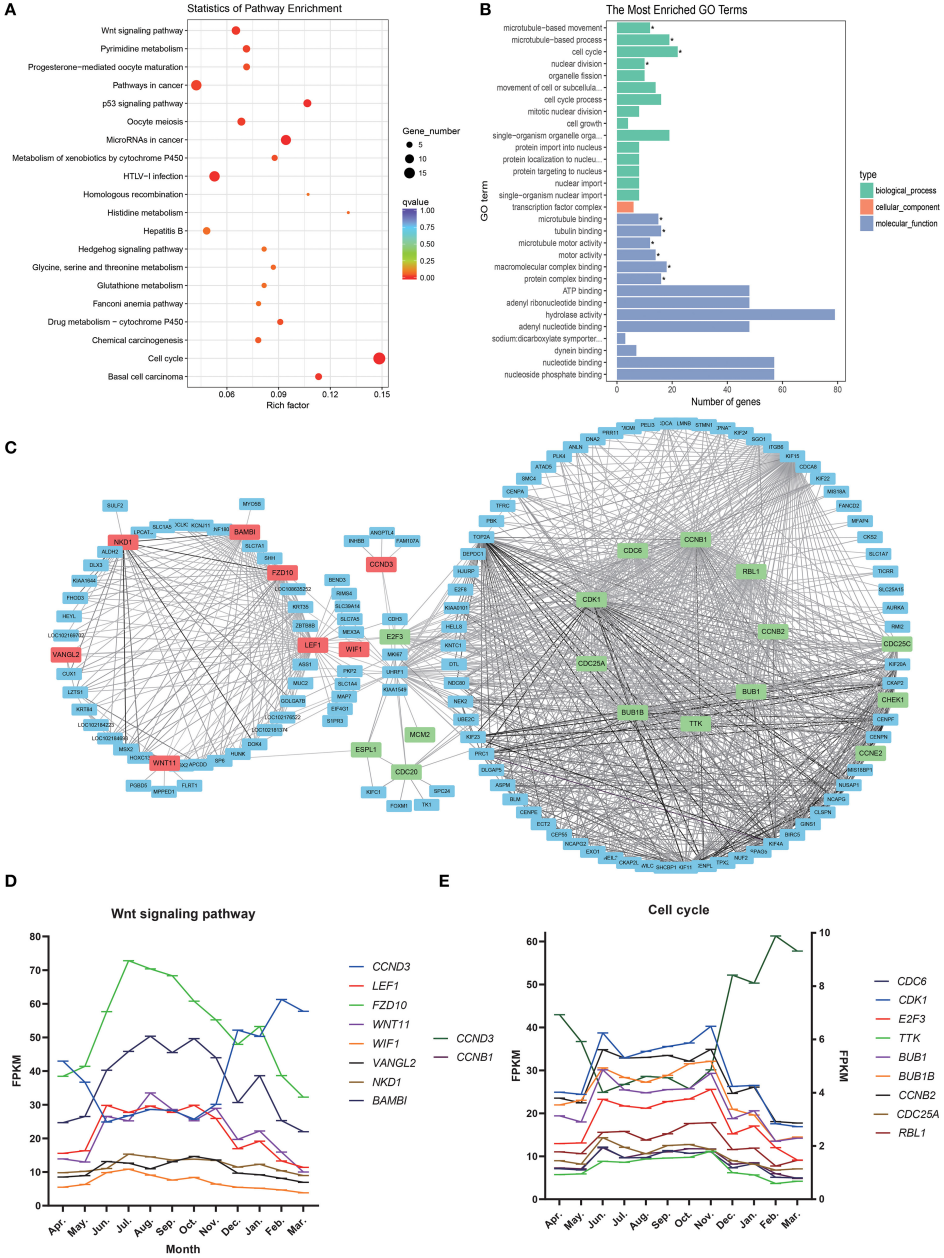
Steelblue module gene analysis map. **(A)** KEGG enrichment analysis of steelblue module. **(B)** GO analysis of steelblue module. **(C)** Gene co-expression network in steelblue modules. **(D)** Gene expression trend of wnt signaling pathway, Abscissa indicates month, in which the anagen (April–November), catagen (December–January) and telogen (February–March), the ordinate is FPKM. **(E)** Gene expression trend of cell cycle, *CCNB*, and *CCND3* use the left ordinate axis, other genes use the left coordinate axis.

In enrichment analysis of KEGG pathways (Figure 4A; Supplementary Table 4), steelblue module genes were significantly enriched in 13 signal pathways, including Cell cycle, Wnt signaling pathway, p53 signaling pathway, pyrimidine metabolism, and drug metabolism-cytochrome P450. Wnt signaling pathway and Cell cycle are typical pathways that regulate the periodic development of hair follicles. Through GO functional enrichment analysis (Figure 4B; Supplementary Table 5). We found that the module genes were significantly enriched in 10 GO annotations, including four biological processes and six molecular functions. The biological processes of enrichment include cell cycle (GO:0007049), microtubule-based movement (GO:0007018), microtubule-based process (GO:0007017), and nuclear division (GO:0000280). The enrichment molecular functions include microtubule binding (GO:0008017), tubulin binding (GO:0015631), microtubule motor activity (GO:0003777), and motor activity (GO:0003774), macromolecular complex binding (GO:0044877) and protein complex binding (GO:0032403).

We constructed a gene network for steelblue module (Figure 4C) and found that genes of the Wnt signaling pathway and Cell cycle pathway were in the center of a network. *LEF1, VANGL2, BAMBI, WNT11, FZD10, NKD1*, and *WIF1* in the Wnt signaling pathway had high connectivity. *CCND3* and *E2F3* belonged to both the Wnt signaling pathway and cell cycle. *CDK1, CCNB1, TTK, BUB1, BUB1B, CCNB2, CDC6, CCND3, CDC25A, RBL1*, and other genes in the Cell cycle were also located in the center of the network, and these candidate genes might play an important regulatory role. In the gene expression trend map of these two pathways (Figures 4D,E), we found that the expression trend of *CCND3* was opposite to that of other genes in the two pathways. The expression of *CCND3* decreased gradually in anagen and began to increase in catagen and telogen. Gene expression trends of other genes in the Wnt signaling pathway and Cell cycle were the same, which increased gradually during anagen, reached the highest level from August to October, then decreased gradually, and continued to decrease in catagen and telogen. Expression levels of most genes in the cell cycle were low.

### Detection of Relative Expression of Candidate Genes by qRT-PCR

**Figure 5:**
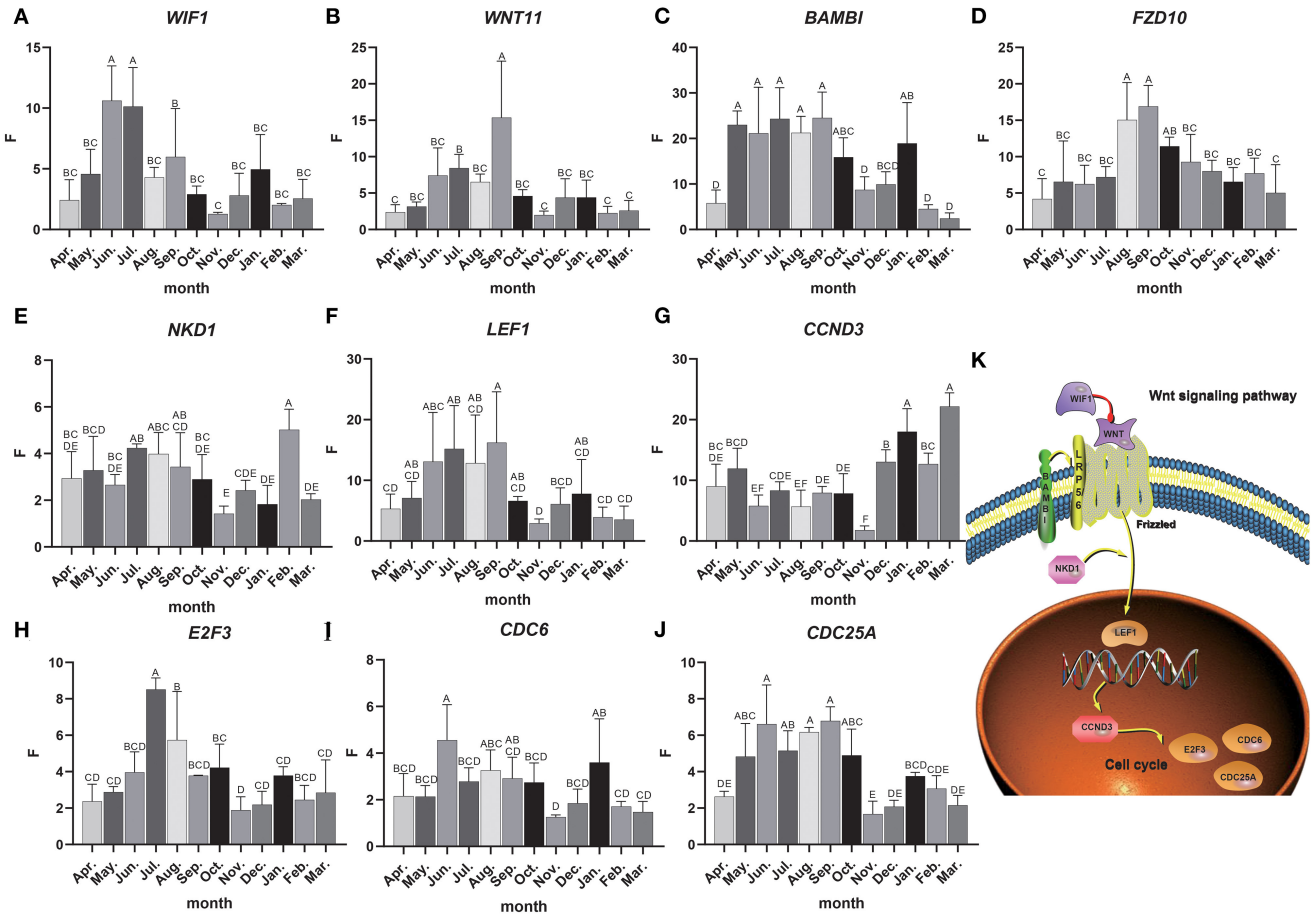
Candidate gene expression analysis. **(A–J)** The relative expression of candidate genes, the Abscissa indicates the month, the ordinate is the relative expression F, different letters are marked to indicate significant, while the same letters indicate that the differences are not significant, the error bar represents SD. **(K)** Interactive network control chart.

Total RNA was extracted from 36 skin samples for 12 months. The concentration was above 180 ng/μl, and OD260/280 was between 1.80 and 2.01. A number of candidate genes related to the periodic development of hair follicles were identified by modular gene analysis. 10 genes (*WIF1, WNT11, BAMBI, FZD10, NKD1, LEF1, CCND3, E2F3, CDC6*, and *CDC25A*) participating in Wnt signaling pathways and cell cycle were selected and validated by qRT-PCR during 12 months (Figures 5A–J). Through multiple comparisons, it was found that the differences between these candidate genes in different months were significant (*p* < 0.05), and a statistical table was made (Table 2).

**Table 2:** Statistical table of relative expression of candidate genes.

**Gene name**	**Anagen**								**Catagen**		**Telogen**		***p-*value**
	**Apr**.	**May**.	**Jun**.	**Jul**.	**Aug**.	**Sep**.	**Oct**.	**Nov**.	**Dec**.	**Jan**.	**Feb**.	**Mar**.	
*WIF1*	2.41 ± 1.69^BC^	4.56 ± 2.04^BC^	10.61 ± 2.87^A^	10.14 ± 3.21^A^	4.30 ± 0.82^BC^	5.99 ± 3.98^B^	2.90 ± 0.68^BC^	1.28 ± 0.13^C^	2.80 ± 1.83^BC^	4.96 ± 2.86^BC^	2.02 ± 0.12^BC^	2.55 ± 1.58^BC^	*P=0.0001*
*WNT11*	2.39 ± 1.03^C^	3.15 ± 0.64^BC^	7.41 ± 3.80^BC^	8.42 ± 1.91^B^	6.53 ± 1.09^BC^	15.36 ± 7.76^A^	4.59 ± 0.87^BC^	1.99 ± 0.55^C^	4.39 ± 2.59^BC^	4.40 ± 2.38^BC^	2.27 ± 0.91^C^	2.60 ± 1.39^C^	*P=0.0003*
*BAMBI*	5.78 ± 2.89^D^	22.99 ± 3.08^A^	21.16 ± 10.1^A^	24.31 ± 6.83^A^	21.25 ± 3.61^A^	24.51 ± 5.68^A^	15.88 ± 4.28^ABC^	8.72 ± 2.88^D^	9.92 ± 2.77^BCD^	18.92 ± 8.97^AB^	4.51 ± 0.96^D^	2.41 ± 1.24^D^	*P <0.001*
*FZD10*	4.20 ± 2.79^C^	6.56 ± 5.61^BC^	6.25 ± 2.58^BC^	7.21 ± 1.44^BC^	15.05 ± 5.13^A^	16.90 ± 2.89^A^	11.44 ± 1.25^AB^	9.28 ± 3.77^BC^	8.00 ± 1.52^BC^	6.56 ± 1.95^BC^	7.71 ± 2.08^BC^	5.03 ± 3.88^C^	*P=0.0011*
*NKD1*	2.93 ± 1.15^BCDE^	3.29 ± 1.45^BCD^	2.66 ± 0.44^BCDE^	4.24 ± 0.18^AB^	3.99 ± 0.92^ABC^	3.43 ± 1.47^ABCD^	2.90 ± 1.06^BCDE^	1.43 ± 0.32^E^	2.42 ± 0.43^CDE^	1.83 ± 0.80^DE^	5.02 ± 0.88^A^	2.03 ± 0.25^DE^	*P=0.0017*
*LEF1*	5.30 ± 2.44^CD^	7.09 ± 2.72^ABCD^	13.08 ± 8.11^ABC^	15.19 ± 7.12^AB^	12.83 ± 7.93^ABCD^	16.20 ± 8.39^A^	6.62 ± 0.71^ABCD^	2.93 ± 0.69^D^	6.10 ± 2.67^BCD^	7.79 ± 5.64^ABCD^	3.92 ± 1.66^CD^	3.54 ± 2.22^CD^	*P=0.0267*
*CCND3*	9.01 ± 3.65^BCDE^	11.94 ± 3.33^BCD^	5.80 ± 1.80^EF^	8.34 ± 1.42^CDE^	5.68 ± 2.71^EF^	7.93 ± 1.05^DE^	7.84 ± 3.26^DE^	1.78 ± 0.71^F^	13.04 ± 2.00^B^	18.01 ± 3.80^A^	12.69 ± 1.79^BC^	22.16 ± 2.25^A^	*P <0.0001*
*E2F3*	2.37 ± 0.94^CD^	2.88 ± 0.30^CD^	3.96 ± 1.12^BCD^	8.51 ± 0.64^A^	5.73 ± 2.67^B^	3.78 ± 0.03^BCD^	4.22 ± 1.29^BC^	1.89 ± 0.74^D^	2.19 ± 0.73^CD^	3.79 ± 0.48^CD^	2.45 ± 0.79*B*^CD^	2.85 ± 1.80^CD^	*P <0.0001*
*CDC6*	2.15 ± 0.97^BCD^	2.14 ± 0.47^BCD^	4.56 ± 1.51^A^	2.78 ± 0.59^BCD^	3.27 ± 0.87^ABC^	2.91 ± 0.91^ABCD^	2.74 ± 0.83^BCD^	1.26 ± 0.09^D^	1.85 ± 0.60^BCD^	3.60 ± 1.87^AB^	1.71 ± 0.22^CD^	1.48 ± 0.45^CD^	*P=0.0073*
*CDC25A*	2.63 ± 0.30^DE^	4.83 ± 1.82^ABC^	6.61 ± 2.15^A^	5.16 ± 1.09^AB^	6.17 ± 0.25^A^	6.79 ± 0.77^A^	4.89 ± 1.44^ABC^	1.67 ± 0.72^E^	2.08 ± 0.35^DE^	3.76 ± 0.21^BCD^	3.07 ± 0.70^CDE^	2.16 ± 0.54^DE^	*P <0.0001*

*The data are expressed in the form of mean ± SD deviation, and p-value is the result of the analysis of ANOVA of genes in different months*.

**ABCDEF:** *Represent significant is significant with different letters (p < 0.05)*.

We found that the quantitative results were basically consistent with transcriptome data, but were different among genes. For instance, the expression level of *WIF1* was higher in anagen, and *WIF1* expression in June and July was significantly higher than that in other months (Figure 5A). The expression of *WNT11* in September was significantly higher than that in other months, while the expression level was the lowest in the catagen and telogen (Figure 5B). The expression of *BAMBI* was the highest from May to September and the lowest during the telogen (Figure 5C). Expression of *FZD10* was the highest in August and September (Figure 5D). The overall expression of *NKD1* was consistent in each month, and the expression level was up-regulated in the anagen (Figure 5E). Expression of *LEF1* was the highest from June to September, and the expression level was lower in the catagen and telogen (Figure 5F). The expression level of *CCND3* was low in anagen, significantly lower in November than in other months, and gradually increased in catagen and telogen (Figure 5G). Expression of *E2F3* was the highest in July and August, and the expression level was lower in the catagen and telogen (Figure 5H). Expression of *CDC6* was consistent in each month (Figure 5I). The expression level of *CDC25A* was higher in the whole anagen, and the expression level from May to October was significantly higher than that in other months (Figure 5J). The expression of these genes changes over time, indicating close correlations to the secondary hair follicle cycle.

Dynamic regulatory network control chart according to the regulatory relationship between Wnt signaling pathway and cell cycle pathways and the expression of candidate genes (Figure 5K). We can find that *WIF1* regulates *WNT11, WNT11*, and *BAMBI* to activate the receptor Frizzled in the Wnt signaling pathway, and then activates *LEF1* under the regulation of the *NKD1* gene, and then inversely regulates *CCND3*. At the same time, it affects *E2F3, CDC6, CDC25A*, and other genes in the cell cycle pathway. These genes may work together to regulate the periodic growth of the hair follicles.

## Discussion

WGCNA is a systematic method to discover functional modules by phenotype-transcriptome joint analysis, function enrichment analysis, reduction of gene dimension, and biological significance analysis of modules. In this study, based on the gene expression data and phenotypic data of Inner Mongolia cashmere goat for 12 months, 7,320 effective differential genes were divided into modules by WGCNA, and a total of 17 co-expression modules were obtained. In order to mine the modules related to hair follicle cycle development, we identified that the steelblue module had the highest correlation with the hair follicle cycle.

The genes in Steelblue modules were significantly enriched in the Wnt signaling pathway and cell cycle, which are typical cycle-related genes. The Wnt signaling pathway is involved in the regulation of development, wound healing, disease and cancer. The Wnt pathway regulates cell proliferation in the skin, which directly affects skin regeneration of wounds. Hair follicles of cashmere goats are also undergoing continuous reborn. Studies have found that the origin of new hair follicle cells is regulated by the Wnt signal pathway to activate hair follicle stem cells (HFSC) (40). The activation of the Wnt signaling pathway can induce HFSC from the telogen to the anagen, thus accelerating hair regeneration and development (20). In this study, we also found that the expression level of genes in Steelblue module began to increase gradually during anagen, and decreased gradually in catagen and telogen. The expression pattern of these genes was consistent with the cycle of hair follicles, which may be involved in the regeneration of hair follicles. Through Figures 4D,E, we can also find that the genes in these two pathways also have the same expression trend.

*WIF1* belongs to the family of Wnt inhibitors, which interferes with Wnt signal transduction by directly binding to WNT proteins (41). The expression of *WIF1* increased in the early stage of hair follicle growth (42), which was consistent with the results of this study. It is known that the expression of *WNT11* is up-regulated during skin wound healing, indicating that the signal transduction of this gene may be active in the stage of wound healing and play an important role in the regeneration of hair follicles (43). *BAMBI* is an inhibitor of TGF-β family members and plays a role in hair follicle development (44). *BAMBI* is different in fetal skin, and the expression from 45 days to 135 days increases gradually in the embryonic stage, which is consistent with the development of hair follicles in the embryonic stage and can positively regulate the growth of hair follicles (45). *BAMBI* also plays an important role in the periodic growth of hair follicles, and it can be found that the expression of *BAMBI* in the whole anagen is significantly higher than that in catagen and telogen, indicating that the expression of *BAMBI* plays a positive role in the rapid maturation of hair follicles during hair follicle growth. The expression of *FZD10* is up-regulated during the transition from the resting stage to the growing stage, which contributes to the activation of HFSC and promotes the circulation and regeneration of hair follicles (46). *LEF1* is a downstream factor of the Wnt signaling pathway and is expressed in inner and outer root sheath cells, hair stromal cells, and dermis. *LEF1* is also highly expressed during the growth period and can promote the growth and development of hair (47). This study has the same results. At the same time, some studies have shown that the expression of *LEF1* in the dermis can support the new development of hair follicles in the wound, induce the expression of *LEF1* in human dermal cells to enhance the ability of skin repair, and regenerate new hair follicles in the healed wound (48). *LEF1* plays an important role in the regulation of hair follicle regeneration. *CCND3* plays an important role in cell proliferation and apoptosis affects the normal progress of the cell cycle and is a key molecule in the process of transformation. *CCND3* knockdown induces cell cycle arrest and apoptosis (49). This study found that the expression level of *CCND3* was higher in the catagen and telogen and the expression of *CCND3* may promote the apoptosis of hair follicle cells and enter the next cycle. *CDC6* encodes key proteins of DNA replication and is responsible for recruiting MCM helicases to the origin of replication during the G1 phase of the cell division cycle. The overexpression of *CDC6* protein in the skin can prolong the retrogression and resting period in the hair growth cycle and promote the preservation of hair (50).

Through this study, it was found that these candidate genes were differentially expressed at different stages of the hair follicle growth cycle. The expression of these genes *WIF1, BAMBI, LEF1, CDC25A, WNT11, FZD10*, and *E2F3* was significantly high in anagen, but low in catagen and telogen, indicating that it plays an important role in the rapid growth and development of hair follicles. The expression of *CCND3* was significantly higher in catagen and telogen, indicating that the gene can promote the apoptosis and regeneration of hair follicles. *NKD1* and *CDC6* showed significant differences only in some months. These findings suggest that these candidate genes may play an important role in the development of secondary hair follicles.

## Conclusions

In this study, we used the WGCNA method to study the important regulatory genes of secondary hair follicle development in cashmere goats and obtained 17 co-expression modules. A strong correlation between Steelblue and multiple stages of hair follicle growth and development was discovered, and identified as a key module. Wnt signaling pathway and Cell cycle play an important role in the periodic development of hair follicles. Ten important genes related to the hair follicle cycle were identified and further verified, including *WIF1, WNT11, BAMBI, FZD10, NKD1, LEF1, CCND3, E2F3*, and *CDC6*. These findings lay a foundation for further study of molecular mechanisms in biological functions such as hair follicle development and hair growth in cashmere goats.

## Data Availability Statement

The transcriptome dataset used in this study can be found in the SRA database and is expected to be made public on June 30, 2024 with the login number PRJNA832904. Other data sets during the current study are available from the corresponding author on reasonable request.Requests to access these datasets should be directed to RS: suruiyu@126.com.

## Ethics Statement

In this study, skins were collected in accordance with the International Guiding Principles for Biomedical Research involving animals and approved by the Special Committee on Scientific Research and Academic Ethics of Inner Mongolia Agricultural University, responsible for the approval of Biomedical Research Ethics of Inner Mongolia Agricultural University [Approval No. (2020) 056].

## Author Contributions

RS, JL, and QL conceived the study. GG, XiaomY, HL, YS, and LZ collected the samples and extracted RNA. GG, WL, JZ, and XiaocY analyzed the data. RS and GG prepared the manuscript. All authors listed have made a substantial, direct, and intellectual contribution to the work and approved it for publication.

## Funding

This work was financially supported by National Natural Science Foundation of China (31860637), China Agriculture Research System of MOF and MARA (CARS-39), Science and Technology Major Project of Inner Mongolia Autonomous Region (2021ZD0012), Scientific Project of Inner Mongolia Agricultural University on High-level Introduced Talented Personnel (NDYB2018-1), Central Government Guides Local Science and Technology Development Fund Projects (2020ZY0007), and Special Funding Project for the Iconic Achievements of the College of Animal Science, Inner Mongolia Agricultural University (No. BZCG202111).

## Conflict of Interest

The authors declare that the research was conducted in the absence of any commercial or financial relationships that could be construed as a potential conflict of interest. The reviewer WB declared a shared affiliation with the author YF to the handling editor at the time of the review.

## Publisher's Note

All claims expressed in this article are solely those of the authors and do not necessarily represent those of their affiliated organizations, or those of the publisher, the editors and the reviewers. Any product that may be evaluated in this article, or claim that may be made by its manufacturer, is not guaranteed or endorsed by the publisher.

## Supplementary Material

Supplementary Table 1 Primer sequences of candidate gene and reference genes.

Supplementary Table 2 Power information statistics table.

Supplementary Table 3 Steelblue module gene information statistical table.

Supplementary Table 4 KEGG enrichment analysis table of steelblue module.

Supplementary Table 5 GO analysis table of steelblue module.
